# Abnormalities in the Fractional Amplitude of Low-Frequency Fluctuation and Functional Connectivity in Parkinson's Disease With Excessive Daytime Sleepiness

**DOI:** 10.3389/fnagi.2022.826175

**Published:** 2022-07-05

**Authors:** Yuheng Zi, Sainan Cai, Changlian Tan, Tianyu Wang, Qin Shen, Qinru Liu, Min Wang, Junli Li, Lin Zhang, Fan Zhou, Chendie Song, Jiaying Yuan, Yujing Liu, Jun Liu, Haiyan Liao

**Affiliations:** ^1^Department of Radiology, The Second Xiangya Hospital, Central South University, Changsha, China; ^2^Department of Radiology, The First Affiliated Hospital, Hengyang Medical School, University of South China, Hengyang, China; ^3^Department of Radiology, Affiliated Hangzhou First People's Hospital, Zhejiang University School of Medicine, Hangzhou, China; ^4^Clinical Research Center for Medical Imaging in Hunan Province, Changsha, China

**Keywords:** Parkinson's disease, excessive daytime sleepiness (EDS), fractional amplitude of low-frequency fluctuation, functional connectivity (FC), resting-state functional MRI (rs-fMRI)

## Abstract

**Background:**

Excessive daytime sleepiness (EDS) is one of the most important non-motor symptoms of Parkinson's disease (PD), and its neuropathologic basis is still unclear.

**Objective:**

This study investigated the changes of neuronal activity in PD patients with EDS (PD-EDS) in the resting state.

**Methods:**

Forty-three PD patients were recruited and divided into the PD-EDS group (*n* = 21) and PD-NEDS group (PD patients without excessive daytime sleepiness, *n* = 22) according to the Epworth sleepiness scale (ESS) scores. Patients in both groups received resting-state functional magnetic resonance imaging (rs-fMRI). The differences in fractional amplitude of low-frequency fluctuation (fALFF) between the two groups, correlations between fALFF and ESS, and functional connection (FC) between the brain regions with different fALFF values and the whole brain were analyzed.

**Results:**

PD-EDS patients exhibited a decreased fALFF in the Cingulum-Ant-R, but an increased fALFF in the Putamen-R and Thalamus-L when compared with PD-NEDS patients; an increased functional connectivity between these three seed regions with different fALFF values and the right medial frontal gyrus, bilateral superior temporal gyrus, left insular, and right precuneus was observed (*p* < 0.05), but a deceased functional connectivity between these three seed regions and the right cerebellum anterior lobe/right brainstem, right middle temporal gyrus and inferior temporal gyrus, right hippocampus/parahippocampal gyrus, right medial cingulate gyrus and bilateral middle occipital gyrus was observed (*p* < 0.05). The value of fALFF was negatively correlated with the ESS score in the Cingulum-Ant-R, but positively correlated with the ESS score in the Putamen-R and Thalamus-L.

**Conclusions:**

EDS in PD patients may be associated with changes in brain neuron activity and functional connectivity.

## Introduction

Excessive daytime sleepiness (EDS) is one of the most important non-motor symptoms of Parkinson's disease (PD). It mainly manifests as an inability to maintain wakefulness and alertness during daytime, while also encompassing an inappropriate sleepiness behavior that includes accidentally succumbing drowsiness (Shen et al., 2018). These symptoms seriously affect the quality of life for PD patients, and they may even increase risks such as traffic accidents for these individuals (Knie et al., 2011). As reported, EDS affects up to 50% of PD patients, with an increase during the progress of the disease (Salawu and Olokoba, 2015; Tholfsen et al., 2015). Previous studies have taken this sleep disorder as a pre-clinical and pre-motor sign of PD and suggest that this symptom can predict the development of PD motor symptoms (Pont-Sunyer et al., 2015; Salawu and Olokoba, 2015). Additionally, studies have found that PD patients with EDS (PD-EDS) have more significant nocturnal disturbances and more severe non-motor symptoms than PD patients without EDS (Yoo et al., 2019). Moreover, EDS has been demonstrated to have a negative impact on non-motor symptoms such as mood, anxiety, and cognitive function (Chan et al., 2020). Therefore, EDS is proposed to play a predictive role in the prognosis of PD. However, the diagnosis of EDS is difficult due to its onset being relatively hidden, and that it overlaps with other non-motor symptoms of PD. Moreover, the neuropathological mechanism of PD-EDS is still unknown; thus, it is important to understand its mechanisms and find diagnostic markers for EDS in PD.

Previous imaging studies have revealed that PD patients with EDS have certain changes in brain function, structure, and metabolism, including regional brain area metabolic activity, neuronal spontaneous activity, brain function connectivity, gray matter volume, white matter fiber tracts, and dopamine transporter metabolism; however, there are still inconsistencies in these conclusions (Gama et al., 2010; Kato et al., 2012; Chondrogiorgi et al., 2016; Wen et al., 2016; Yousaf et al., 2018; Ashraf-Ganjouei et al., 2019; Yoo et al., 2020). The reasons include the heterogeneity of samples and diseases, the inconsistency of data analysis and statistical methods, and so on. Most previous studies focused on the neural mechanism of PD-EDS from the perspective of functional differentiation using a single-modal, so they could not fully explain EDS' pathogenesis.

Resting-state functional magnetic resonance imaging (RS-fMRI) is a widely used, non-invasive technique. Many data analysis methods based on RS are emerging. Among them, the fraction amplitude of low-frequency fluctuation (fALFF) method can reduce the interference of physiological noise to a certain extent and reduce the influence of non-neuronal activity-related factors. Thus, the detected signals are more sensitive and specific (Zou et al., 2008). The functional connectivity (FC) method analyzes the correlation and synchronization of spontaneous activities of the selected seed point and other voxels of the whole brain. The fALFF and FC methods reflect the spontaneous activities and integrated functions of brain neurons from the local and overall brain levels, which are helpful to analyze the neuroimaging changes of PD-EDS from multiple aspects. Therefore, this study analyzed the changes of fALFF and FC in PD patients with EDS, and their correlations with clinical indicators, in order to explore the pathogenesis of EDS in PD. Changes in brain functions provide imaging evidence for the occurrence and development of EDS in PD.

## Materials and Methods

### Participants

**Figure 1:**
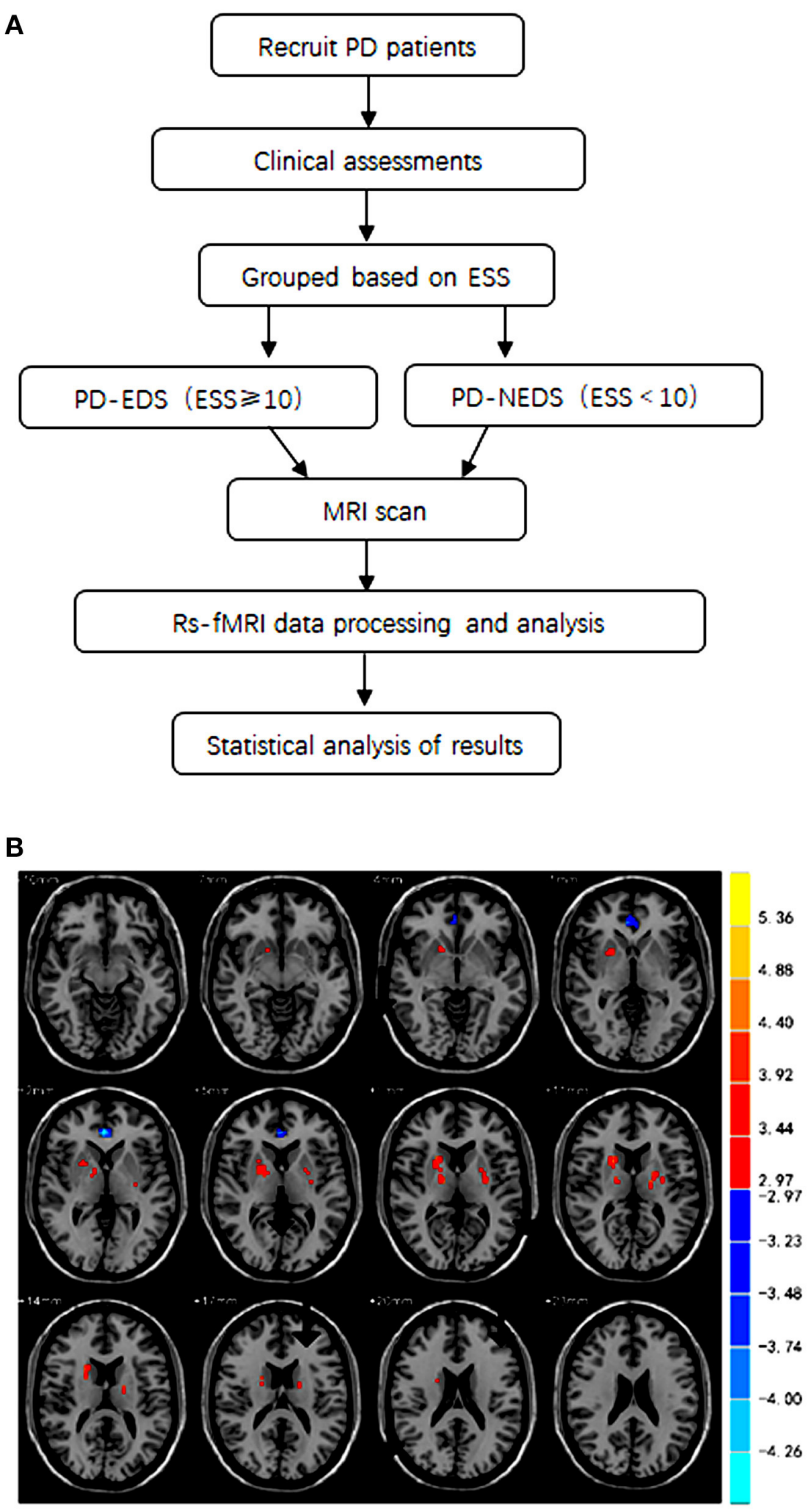
**(A)** A flowchart of the collection of patients and study design. **(B)** The brain areas with statistically significant differences in fALFF values between the PD-EDS and PD-NEDS group (*P* < 0.05, AlphaSim corrected).

A total of 43 PD patients were recruited from the Department of Neurology, the Second Xiangya Hospital of Central South University during the period between June 2018 and November 2020. A flowchart of the collection of patients and study design was provided in Figure 1A. This study was approved by the Medical Ethics Committee of the hospital, and all subjects signed informed consent to participate in the study voluntarily. Patients were enrolled in the study if they met the following inclusion criteria: (1) patients met the 2015 diagnostic criteria of the International Movement Disorder Society; (2) patients were right-handed; and (3) patients completed the evaluation of all clinical scales and the whole process of functional MR sequence scanning. The exclusion criteria were as follows: (1) patients had intracranial organic lesions, such as tumor, hematoma, cerebral infarction, etc.; (2) patients had Parkinson's syndrome or Parkinson's superimposed syndrome caused by other definite diseases; (3) patients had previous traumatic brain injury; (4) patients took drugs that interfere with sleep, such as hypnotics; (5) patients had contraindications of MRI, and (6) patients took sleep-disrupting medicine.

The participants were divided into two groups according to Epworth sleepiness scale (ESS) scores; the Epworth sleepiness scale was used to assess sleep symptoms in the enrolled Parkinson's patients. Twenty-one PD patients with an ESS score of 10 or greater were placed into the PD-EDS group, and twenty-two PD patients with an ESS score <10 were placed into the PD-NEDS group.

### Clinical Assessment

All patients were diagnosed by a neurologist. Patients' age, condition, and course of disease—as well as other related information—were recorded. All patients were evaluated by comprehensive clinical assessments including motor and non-motor symptom related scales, such as the United Parkinson's disease rating scale (UPDR), part III of the unified Parkinson's disease rating scale (UPDRS-III), the Hoehn and Yahr (H-Y) scale, the Mini-mental state examination (MMSE), the Hamilton depression scale (HAMD), and the Epworth sleepiness scale (ESS).

### MRI Acquisition

All patients stopped taking antiparkinsonian medications more than 12 hours before scanning. The subjects were instructed to keep quiet and close their eyes during scanning, but to stay awake without movement and thinking activity. Routine sequence scanning was first performed to exclude other intracranial organic lesions. The head MRI images of all subjects were scanned by a skilled technician, on a Siemens skyra 3.0T MRI machine with sixteen-channel head coil. The sequence scanning parameters of T1-weighted three-dimensional magnetization-prepared rapid gradient echo were as follows: repetition time = 1,900 ms, echo time = 2.01 ms, field of view = 256 mm × 256 mm, slice thickness = 1.0 mm, slices = 176, flip angle = 9°, matrix size = 256 × 256. The sequence scanning parameters of resting-state echo-planar imaging were as follows: repetition time = 2,500 ms, echo time = 25 ms, flip angle = 90°, field of view = 240 mm × 240 mm, matrix size = 64 × 64, slice thickness = 3.5 mm, slices = 39, interval = 0 mm.

### Data Processing

MRIcro software was used to convert the original image data from DICOM format to NIFTI format. The rest-plus toolkit based on MATLAB 2014a software was used to preprocess and analyze the data. The main steps were as follows: (1) eliminating the first 10 time points of the initial signal; (2) using slice timing correction; (3) excluding the data of subjects with excessive head movement (horizontal movement > 0.5 mm or rotation angle >0.5°); (4) co-registration based on T1 structural image and spatial normalization to Montreal Neurological Institute coordinates (MNI) space; (5) smoothing; (6) linear detrending; (7) applying nuisance covariates regression (head movement parameters); and (8) extracting low frequency (0.01 Hz < f < 0.08 Hz) signal.

### fALFF Calculation

The rest-plus toolkit was used to calculate the fractional amplitude of low-frequency fluctuations (fALFF) of the preprocessed data. The fALFF is the ratio of the amplitude of each frequency in the low frequency range (0.01–0.08 hz) to the amplitude of the whole frequency range (0–0.25 Hz) (Zou et al., 2008). The value was divided by the average fALFF value of the whole brain and standardized to obtain the standardized fALFF value of each voxel.

### FC Calculation

Regions showing significant fALFF differences between PD patients with and without EDS were used as the seed regions. Correlation analysis was conducted between the seed regions and the whole-brain in a voxel-wise manner. The FC values from the correlation analysis were then normalized using Fisher r-to-z transformation. An entire brain z-value map was created for each subject.

### Statistical Analysis

The clinical data were analyzed using SPSS20.0 software. Independent sample *t*-test was used to compare the differences in age, onset age, education level, course of disease, H-Y staging, UPDRS scores, UPDRS-III scores, MMSE scores, and HAMD scores between PD-EDS and PD-NEDS patients. Chi square test was used to compare the gender differences between the two groups. A *p* < 0.05 was considered statistically significant.

Brain regions with significant differences of fALFF values between PD-EDS and PD-NEDS patients were compared using two sample *t*-test with the rest-plus toolkit in MATLAB 2014a software. The significant difference was set at *p* < 0.005 before correction (*p* < 0.05 after AlphaSim correction, and cluster size was not <26 voxels). To determine the associations between regions showing fALFF differences and EDS, fALFF differences in these regions were extracted to correlate with ESS scores using Pearson's correlation.

## Results

### Demographic and Clinical Characteristics

No significant differences in age, onset age, gender, education level, and disease duration were observed between the PD-EDS and PD-NEDS group. There were no significant differences in H-Y staging, UPDRS scores, UPDRS-III scores, MMSE scores, and HAMD scores between two groups (*p* > 0.05). PD-EDS patients showed higher ESS scores than PD-NEDS patients (*p* < 0.001) (Table 1).

**Table 1:** Demographic and clinical characteristics of the PD-EDS and PD-NEDS group (Mean ± SD).

	**PD-EDS**	**PD-NEDS**	***P***
Number	21	22	
Gender (M/F)	13/8	9/13	0.227
Age (y)	61.33 ± 10.33	55.95 ± 9.15	0.078
Onset age (y)	57.93 ± 8.85	54.07 ± 8.79	0.159
Disease duration (m)	40.86 ± 40.01	22.65 ± 18.02	0.067
Education (y)	5.43 ± 3.31	6.32 ± 3.76	0.416
H-Y staging	2.00 ± 0.74	1.73 ± 0.59	0.189
ESS sore	13.81 ± 3.09	2.41 ± 2.26	<0.001
UDPRS sore	41.14 ± 22.78	29.91 ± 13.29	0.058
UDPRS-III sore	25.14 ± 16.14	18.45 ± 8.41	0.094
MMSE sore	24.48 ± 3.79	24.27 ± 5.48	0.889
HAMD sore	9.19 ± 6.49	7.55 ± 6.80	0.422

*PD-EDS, Parkinson's disease with excessive daytime sleepiness; PD-NEDS, Parkinson's disease without excessive daytime sleepiness; M, Male; F, Female; y, Year; m, Month; H-Y, Hoehn and Yahr scale; ESS, Epworth Sleepiness Scale; UPDRS, United Parkinson's Disease Rating Scale; UDPRS-III, part III of the Unified Parkinson's Disease Rating Scale; MMSE, Mini-Mental State Examination; HAMD, Hamilton Depression Scale. P < 0.05 was considered significant*.

### fALFF Data

**Figure 2:**
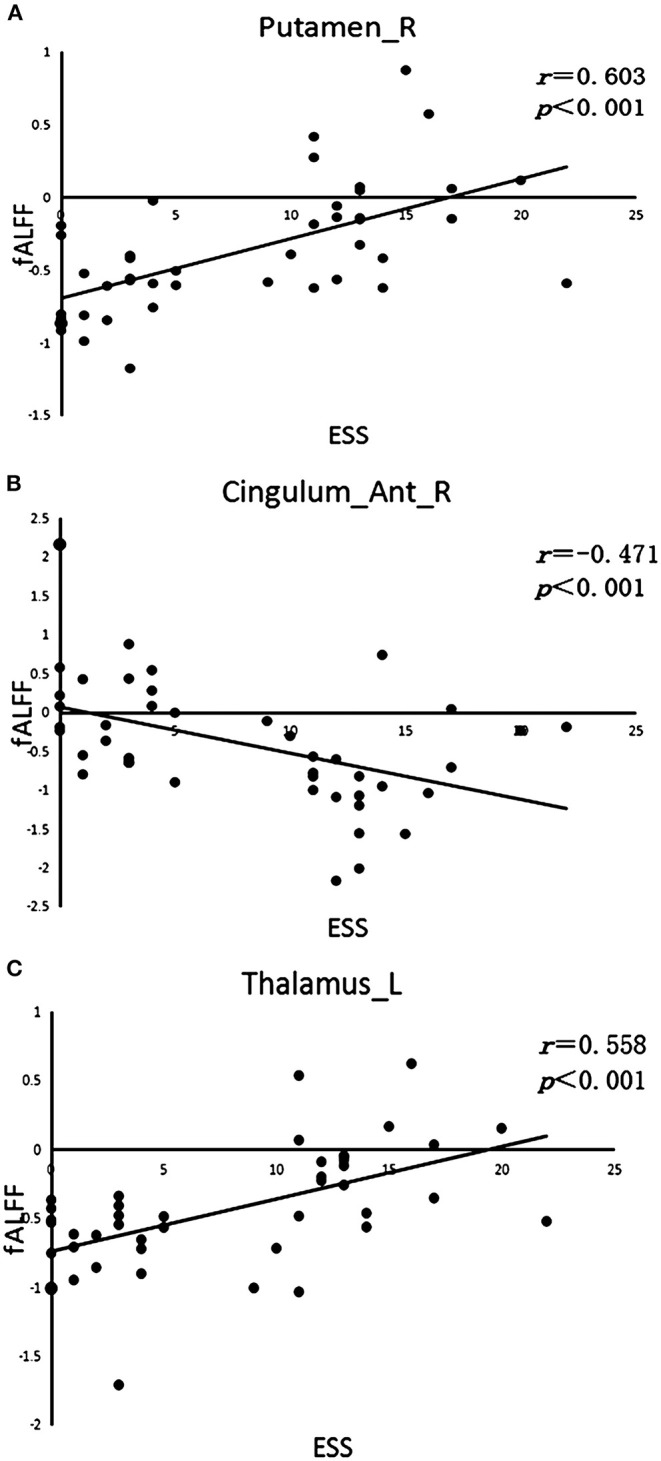
Correlation analysis. **(A)** Correlation between ESS scores and fALFF value in the right putamen (Putamen-R). **(B)** Correlation between ESS scores and fALFF value in the right anterior cingulate gyrus (Cingulum-Ant-R). **(C)** Correlation between ESS scores and fALFF value in the left thalamus (Thalamus-L).

The fALFF analysis showed that PD-EDS patients had a decreased fALFF in the Cingulum-Ant-R, but an increased fALFF in the Putamen-R and Thalamus-L when compared with the PD-NEDS patients (Table 2, Figure 1B). The Pearson correlation analysis showed that ESS scores significantly negatively correlated with fALFF values in the Cingulum-Ant-R (*r* = −0.471, *p* < 0.001) and significantly positively correlated with fALFF values in the Putamen-R (*r* = 0.603, *p* < 0.001) and Thalamus-L (*r* = 0.558, *p* < 0.001) (Figure 2).

**Table 2:** The brain areas with statistically significant differences in fALFF values between the PD-EDS and PD-NEDS group.

**Brain areas (AAL)**	**MNI coordinate**			**Broadmann areas**	**Cluster size**	**T Value**	***P*** **-value**
	**X**	**Y**	**Z**				
Putamen-R	21	9	12	–	65	4.5684	<0.005
Cingulum-Ant-R	3	39	3	32	34	−4.5165	<0.005
Thalamus-L	−18	−12	12	–	26	4.0979	<0.005

### FC Data

**Figure 3:**
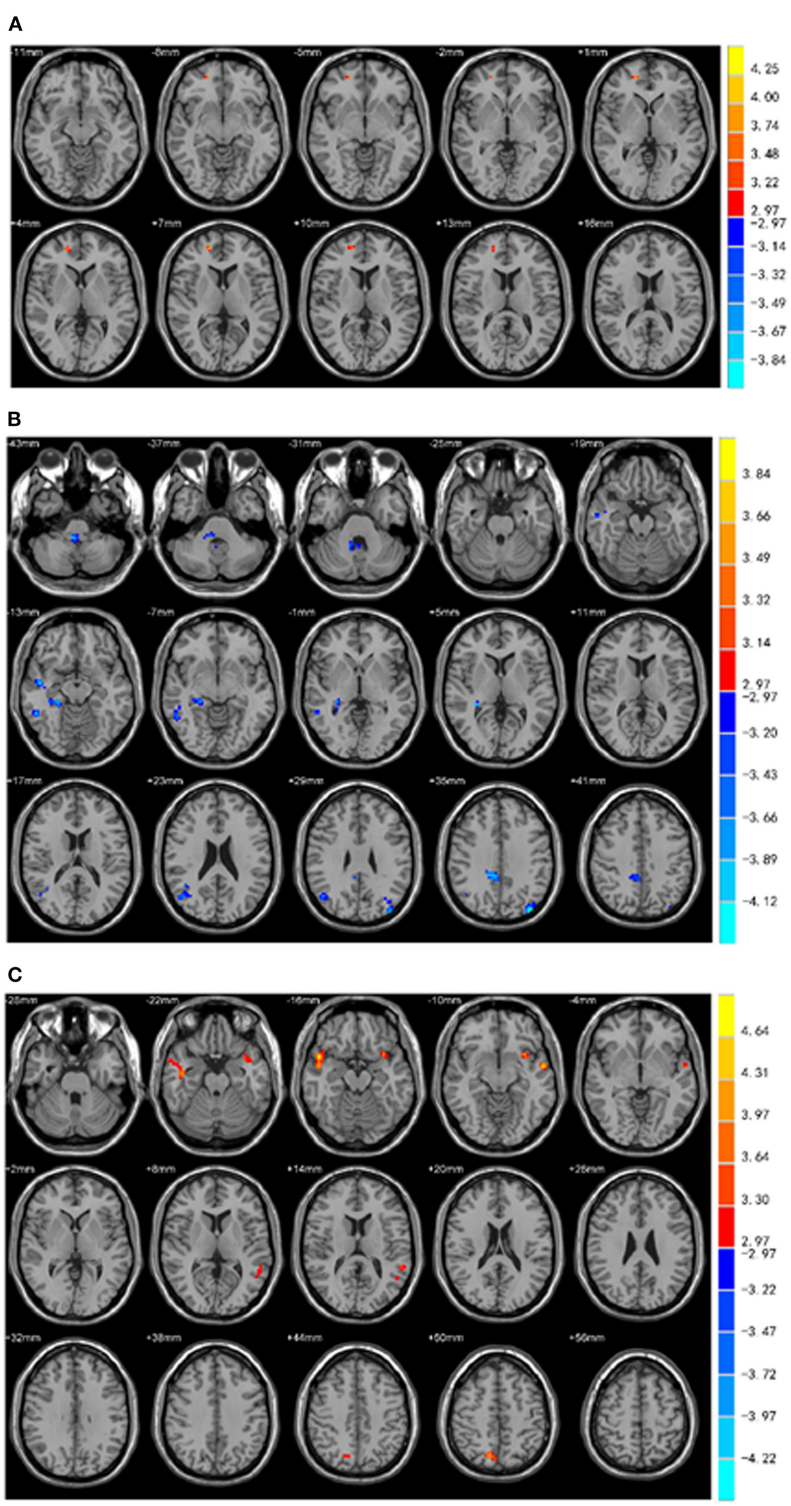
Functional connectivity analysis. **(A)** Using right putamen as the seed point, the FC of the right medial frontal gyrus in the PD-EDS group was increased compared with the PD-NEDS group (red area in the figure) (*P* < 0.05, after AlphaSim correction). **(B)** With the right anterior cingulate gyrus as the seed point, the FC in the right anterior cerebellar lobe/right brainstem, right middle temporal gyrus and inferior temporal gyrus, right hippocampus/para hippocampus, right medial and lateral cingulate gyrus, bilateral occipital middle gyrus was decreased in PD-EDS patientscompared with the PD-NEDS patients (blue area in the figure) (*P* < 0.05, after AlphaSim correction). **(C)** Taking the left thalamus as the seed point, the FC in the bilateral superior temporal gyrus, the left insula, and the right precuneus were increased in PD-EDS patients compared with the PD-NEDS patients (red area in the figure) (*p* < 0.05, After AlphaSim correction).

The three brain regions (Cingulum-Ant-R, Putamen-R, and Thalamus-L) with different fALFF values between the two groups were set as the seed regions. Functional connectivity between the three seeds and the right medial frontal gyrus, bilateral superior temporal gyrus, left insular and right precuneus was increased in PD-EDS patients (*p* < 0.05), whereas the functional connectivity in the right cerebellum anterior lobe/right brainstem, right middle temporal gyrus and inferior temporal gyrus, right hippocampus/parahippocampal gyrus, right medial cingulate gyrus, and bilateral middle occipital gyrus was decreased in PD-EDS patients when compared to PD-NEDS patients (*p* < 0.05) (Table 3, Figure 3).

**Table 3:** Comparison of FC data.

**Brain region**	**MNI**			**BAregion**	**Voxel**	**T-value**	***P-*** **value**
	**X**	**Y**	**Z**				
**Putamen-R as seed**							
Right medial frontal gyrus	18	51	6	–	26	4.0234	<0.005
Cingulum-Ant-R as seed							
Right anterior cerebellar lobe/right brainstem	6	−39	−42	–	62	−3.968	<0.005
Right middle temporal gyrus	48	−9	−12	–	35	−4.1484	<0.005
Right inferior temporal gyrus	54	−48	−12	–	44	−3.8964	<0.005
Right hippocampus/para hippocampus	27	−30	−3	–	64	−4.2481	<0.005
Right middle occipital gyrus	48	−66	27	39	61	−3.8362	<0.005
Left middle occipital gyrus	−36	−84	36	19	47	−4.3471	<0.005
Right medial and lateral cingulate gyrus	12	−39	36	–	61	−3.9835	<0.005
**Left thalamus as seed**							
Right superior temporal gyrus	51	9	−15	–	78	4.7077	<0.005
Left insula	−33	12	−12	13	43	4.9784	<0.005
Left superior temporal gyrus	−57	−3	−9	–	26	4.8294	<0.005
	−57	−42	12	–	33	3.6351	<0.005
Right precuneus	15	−69	48	–	38	4.1807	<0.005

## Discussion

This study, for the first time, compared the resting brain function changes between PD patients with EDS and without EDS using fALFF and seed point-based FC methods. This study found that: (1) The right putamen, left thalamus, and right anterior cingulate gyrus are the key brain regions for EDS in PD patients; (2) Abnormal functional connections of brainstem, limbic system, default network, and cerebellum, etc. were associated with EDS in PD patients.

Sleep is a whole brain status and regulated by interactions between the arousal systems containing the hypothalamus, basal forebrain, and brainstem (Pace-Schott and Hobson, 2002). The occurrence of EDS in PD is related to multiple factors. For instance, the pathological analysis of EDS in PD patients has reported abnormalities in regulating sleep and wakefulness (Shen et al., 2018), the involvements of neurodegeneration in some specific brain regions (Junho et al., 2018), and dopaminergic neuron degeneration in the substantia nigra striatal area (Happe et al., 2007). A previous study suggested that a compensation mechanism of the striatum may be attributed to EDS symptoms in early PD (Gong et al., 2020). Thus, the striatum may be an early biomarker to detect the occurrence of EDS in PD. The putamen is one of the constituent structures of the striatum and an important node of multiple neural circuits and neural networks. Changes in the neural function of the putamen may cause abnormal striatal function and subsequently lead to disorders of sleep cycle regulation. This study found that PD-EDS patients had a lower fALFF value in the right anterior cingulate gyrus, but higher fALFF values in the right putamen and left thalamus when compared to PD-NEDS patients. In these brain areas, fALFF values were correlated with ESS scores. These findings suggest that in PD-EDS patients, the spontaneous activity of neurons is reduced in the right anterior cingulate gyrus, whereas activity is increased in the right putamen and left thalamic neurons, when compared with PD-NEDS patients. Therefore, the occurrence of EDS in PD patients may be related to the neurological down-regulation and compensatory mechanisms in the putamen, thalamus, and anterior cingulate gyrus; the spontaneous changes in neuron activity of the thalamus may disrupt the sleep-wake cycle and lead to the occurrence of EDS. Collectively, the right putamen, left thalamus, and right anterior cingulate gyrus are the key brain areas for EDS in Parkinson's disease.

This study performed a whole brain functional connectivity analysis based on three brain regions, with statistical difference in fALFF between the two groups of patients as the seed points to study the changes in the functional connection within the whole brain. Our study found that the functional connectivity between the three seed points and the right anterior cerebellum/right brainstem, right middle temporal gyrus and inferior temporal gyrus, right hippocampus/para hippocampal gyrus, right medial and para cingulate gyrus, bilateral middle occipital gyrus, etc. was decreased, whereas functional connectivity was increased between seed points and the right medial frontal gyrus, bilateral superior temporal gyrus, left insula, and right precuneus in the PD-EDS patients when compared with PD-NEDS patients. These findings indicate abnormality of integration function in the whole brain of PD patients with EDS.

The thalamus and brainstem are the sleep regulation centers. The brainstem reticulum ascending activation system is the structure of the central nervous system that maintains arousal behavior. When the ascending activation system is inhibited, it causes sleep behavior. In addition to the changes in spontaneous activity of thalamic neurons in PD-EDS patients, the FC results also showed abnormal brainstem functional connectivity in PD-EDS patients. Previous studies have demonstrated that the thalamus is involved in the pathogenesis of PD (Rüb et al., 2002), and a key region for regulating sleep circadian rhythms (Colavito et al., 2015). Moreover, PD patients with abnormal sleep have a general degradation of the brainstem sleep structure (Gama et al., 2010); the impaired brainstem function is the pathogenesis of EDS in PD patients (Tholfsen et al., 2015); and brainstem lesions are proposed as an underlying pathological basis of sleep disorders in PD patients (Pavese, 2014). Collectively, our findings are consistent with previous findings that prove an association between the spontaneous activity of thalamic neurons and EDS in PD patients. Also, the FC results showed a decrease in the brainstem functional connection in PD-EDS patients when compared with PD-NEDS patients, indicating that thalamus and brainstem functional damage is also associated with the occurrence of EDS in PD patients.

The dopamine neurons in the mesencephalic limbic system that project from the ventral tegmental area to the thalamus, hippocampus, and cerebral cortex are thought to be involved in the arousal mechanism (De Keyser et al., 1989). Decreased dopamine in PD may be responsible for excessive daytime sleepiness (Rothman and Mattson, 2012), and any of the above structural disruptions may also disrupt arousal mechanisms. This study found that the functional connectivity between right putamen, left thalamus, and right anterior cingulate gyrus and the right hippocampus and para-hippocampal gyrus was reduced. It is speculated that the functional connectivity of the hippocampus and para-hippocampal gyrus is impaired in PD-EDS patients, which may result in damage to the dopaminergic circuits in the midbrain limbic system and subsequently inhibit the arousal mechanism.

The hippocampus, medial prefrontal lobe, anterior cuneiform lobe, cingulate gyrus and other areas are the key nodes of the default mode network (DMN) (Uddin et al., 2009). A previous study has reported that the occurrence of EDS is related to various factors such as disease course, severity, and cognitive and emotional disorders (Chahine et al., 2017). However, DMN is related to episodic memory, emotion and cognition, and other processes. This study found that the functional connectivity in the right medial frontal gyrus, right anterior cuneiform lobe, right hippocampus, right medial and lateral cingulate gyrus in PD-EDS patients had increased or decreased, and fALFF in the right anterior cingulate gyrus was decreased. Therefore, the results of this study may also implicate that the DMN of PD-EDS patients may be abnormal to a certain extent. We therefore speculated that the occurrence of EDS may be related to DMN. In addition, many imaging studies have shown that EDS is related to DMN. For example, some researchers proposed that there is an inverse relationship between EDS and the functional connection between thalamocortex and DMN (Killgore et al., 2015), and EDS is related to decreased DMN connectivity in young people and elderly people with intact cognition (Ward et al., 2013). A recent study reported a positive relationship between the occurrence of EDS and DMN network connectivity in patients with early PD-EDS, suggesting a compensatory mechanism in the early stage of the disease (Ooi et al., 2019). The results of this study also support the view that the occurrence of EDS is related to abnormal changes in DMN.

In addition, this study also found that the cerebellum is associated with EDS in PD. The FC results show that the functional connection of the right anterior cerebellum is reduced. Recently, some studies have shown that the cerebellum is involved in the occurrence of sleep disorders (Liu et al., 2015), including daytime sleepiness (DelRosso and Hoque, 2014). In addition, Wen et al. (2016) and Ashraf-Ganjouei et al. (2019) study mentioned changes in cerebellar connectivity in PD patients with EDS. Gama et al. (2010) study showed that the occurrence of EDS in PD patients is related to the atrophy of the medial cerebellar foot. Kato et al. (2012) found that the volume of cerebellar gray matter in PD-EDS patients was reduced, and our results are consistent with the findings in the above studies.

This study strictly followed standard procedures in recruiting participants, collecting clinical and magnetic resonance data, and processing and analyzing experimental data, but it still has the following shortcomings: First, this is a cross-sectional study, no follow-up study was conducted, and the dynamic changes of brain functions in patients with PD and EDS were not analyzed; Second, although all subjects stopped taking anti-PD drugs for at least 12 h before receiving the imaging scan, it is not entirely certain whether long-term drug use has a potential impact on the results of the experiment. Third, the sample size of the study is not large, and there may be statistical bias. In addition, this study only analyzed the brain function changes of the two groups of PD patients with EDS and without EDS and did not include the health control group. In future studies, health controls will be included to control confounding factors as much as possible. Large-sample, multi-center, and follow-up studies will help us fully understand the neuroimaging mechanism of EDS in Parkinson's disease.

In conclusion, this study found that PD-EDS patients have changes in the spontaneous activity of neurons and functional connection in multiple different brain regions, such as the putamen, thalamus, anterior cingulate gyrus, brainstem, limbic system, default network, and cerebellum; this reflects that PD-EDS patients have neurological down-regulation or compensation mechanisms in specific areas, as well as abnormal brain integration functions. At the same time, we found that the putamen, thalamus, and anterior cingulate gyrus may be the key brain areas for EDS of Parkinson's disease. This study provides new ideas for further exploring the neuropathological mechanism of EDS in Parkinson's disease.

## Data Availability Statement

The original contributions presented in the study are included in the article/supplementary material, further inquiries can be directed to the corresponding author.

## Ethics Statement

The studies involving human participants were reviewed and approved by the Medical Ethics Committee of The Second Xiangya Hospital. The patients/participants provided their written informed consent to participate in this study.

## Author Contributions

YZ, SC, CT, TW, QS, QL, MW, JLi, LZ, FZ, CS, JY, and YL: data collection and data analysis. YZ and SC: manuscript writing. JLiu and HL: project development and manuscript revising. All authors read and approved the final manuscript.

## Funding

This study was supported by the Natural Science Foundation of Changsha City (No. kp2202416).

## Conflict of Interest

The authors declare that the research was conducted in the absence of any commercial or financial relationships that could be construed as a potential conflict of interest.

## Publisher's Note

All claims expressed in this article are solely those of the authors and do not necessarily represent those of their affiliated organizations, or those of the publisher, the editors and the reviewers. Any product that may be evaluated in this article, or claim that may be made by its manufacturer, is not guaranteed or endorsed by the publisher.
